# Dicarbonyl­dichloridobis(trimethyl­phosphane)iron(II)–carbonyl­dichlorido­tris(trimethyl­phosphane)iron(II)–tetra­hydro­furan (1/1/2)

**DOI:** 10.1107/S1600536811009305

**Published:** 2011-03-19

**Authors:** Nigam P. Rath, Meghan Stouffer, Matthew K. Janssen, John R. Bleeke

**Affiliations:** a Department of Chemistry and Biochemistry and Center for Nanoscience, University of Missouri–St Louis, 1 University Boulevard, St Louis, MO 63121-4400, USA; b Department of Chemistry, Washington University, One Brookings Drive, St Louis, MO 63130-4899, USA

## Abstract

The asymmetric unit of the title crystal, [FeCl_2_(C_3_H_9_P)_3_(CO)]·[FeCl_2_(C_3_H_9_P)_2_(CO)_2_]·2C_4_H_8_O, contains half mol­ecules of the two closely related Fe^II^ complexes lying on mirror planes and a tetra­hydro­furan solvent mol­ecule, one C atom of which is disordered over two sets of sites with site occupancy factors 0.633 (9) and 0.367 (9). In both Fe^II^ complex mol­ecules, a distorted octa­hedral coordination geometry has been observed around the Fe atoms. Weak intermolecular C—H⋯O inter­actions are observed in the crystal structure.

## Related literature

For the synthetic background, see: Harris *et al.* (1978 ▶). For the crystal structure of a related complex, see: Venturi *et al.* (2004 ▶).
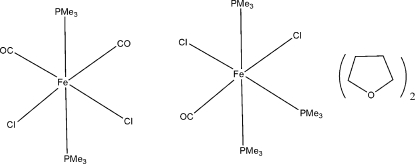


## Experimental

### 

#### Crystal data

[FeCl_2_(C_3_H_9_P)_3_(CO)]·[FeCl_2_(C_3_H_9_P)_2_(CO)_2_]·2C_4_H_8_O
*M* *_r_* = 862.10
Orthorhombic, 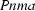

*a* = 10.8391 (9) Å
*b* = 16.9670 (12) Å
*c* = 22.2871 (18) Å
*V* = 4098.8 (6) Å^3^
*Z* = 4
Mo *K*α radiation
μ = 1.20 mm^−1^
*T* = 100 K
0.20 × 0.12 × 0.10 mm

#### Data collection

Bruker APEXII CCD diffractometer
Absorption correction: multi-scan (*SADABS*; Bruker, 2008 ▶) *T*_min_ = 0.800, *T*_max_ = 0.895
144514 measured reflections
4904 independent reflections
3939 reflections with *I* > 2σ(*I*)
*R*_int_ = 0.090

#### Refinement

*R*[*F* ^2^ > 2σ(*F* ^2^)] = 0.034
*wR*(*F* ^2^) = 0.081
*S* = 1.06
4904 reflections
218 parameters
1 restraint
H-atom parameters constrained
Δρ_max_ = 0.68 e Å^−3^
Δρ_min_ = −0.70 e Å^−3^

### 

Data collection: *APEX2* (Bruker, 2010 ▶); cell refinement: *SAINT* (Bruker, 2009 ▶); data reduction: *SAINT*; program(s) used to solve structure: *SHELXS97* (Sheldrick, 2008 ▶); program(s) used to refine structure: *SHELXL97* (Sheldrick, 2008 ▶); molecular graphics: *SHELXTL* (Sheldrick, 2008 ▶) and *Mercury* (Macrae *et al.*, 2008 ▶); software used to prepare material for publication: *SHELXTL* and *PLATON* (Spek, 2009 ▶).

## Supplementary Material

Crystal structure: contains datablocks I, global. DOI: 10.1107/S1600536811009305/pv2394sup1.cif

Structure factors: contains datablocks I. DOI: 10.1107/S1600536811009305/pv2394Isup2.hkl

Additional supplementary materials:  crystallographic information; 3D view; checkCIF report

## Figures and Tables

**Table 1:** Hydrogen-bond geometry (Å, °)

*D*—H⋯*A*	*D*—H	H⋯*A*	*D*⋯*A*	*D*—H⋯*A*
C42—H42*B*⋯O1*S*^i^	0.98	2.53	3.422 (3)	151
C43—H43*C*⋯O1^i^	0.98	2.58	3.510 (3)	158
C43—H43*A*⋯O1^ii^	0.98	2.43	3.392 (3)	167

Symmetry codes: (i) 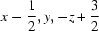; (ii) 
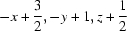
.

**Fig. 1.**
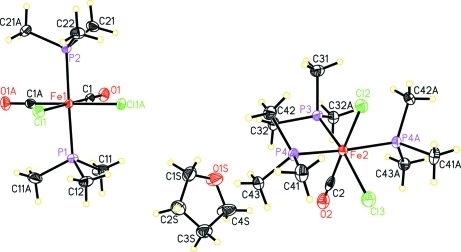
The molecular structure of (I) and (II) with atom labels and 50% probability displacement ellipsoids for non-H atoms. Disordered atoms in the solvent are omitted for clarity. Symmetry codes represented by A in atomic labels: for (I) = x, 0.5 - y, z and for (II) = x, 1.5 - y, z.

**Fig. 2.**
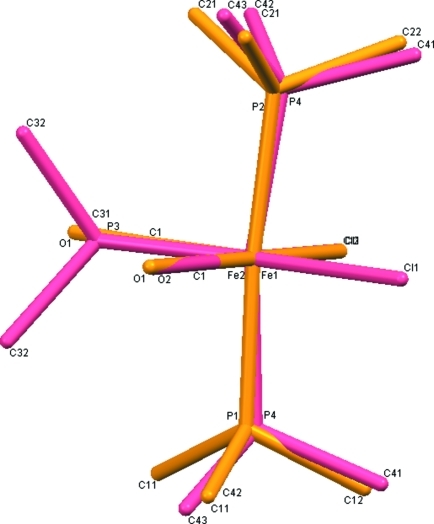
Overlay plot of the two molecules.

**Fig. 3.**
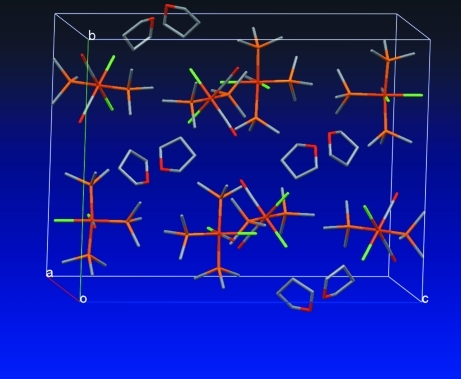
A unit cell packing plot of the title crystal; H atoms have been omitted for clarity.

## supplementary crystallographic information

### Comment

An interesting cocrystallization has occurred from a reaction of CO with Cl_2_Fe(PMe_3_)_2_, resulting in compound (I), C_8_H_18_Cl_2_FeO_2_P_2_, from the addition of two equivalents of CO to Cl_2_Fe(PMe_3_)_2_ and compound (II), C_10_H_27_Cl_2_FeOP_3_, probably from the addition of one equivalent of CO, followed by the rapid addition of one equivalent of free PMe_3_, which is present in the reaction solution. In this paper, we report the crystal structure of the two compounds, (I) and (II) which have been cocrystallized along with a molecule of tetrahydrofuran solvate per molecule of complex (Fig. 1).

The asymmetric unit of the title crystal contains half molecules of the two compounds, (I) and (II), lying on mirror planes and a molecule of tetrahydrofuran solvate, C_4_H_8_O; a carbon atom of the solvent molecule is disordered over two sites C4S and C4S' with site occupancy factors 0.633 (9) and 0.367 (9). In compound (I), the PMe_3_ ligands occupying axial positions, are *trans* with respect to each other with an angle of 175.20 (4)° and the CO and Cl are *trans* with respect to each other at equatorial positions. In compound (II), the *trans* PMe_3_ ligands are located at 166.41 (4)° to each other; the 3^rd^ PMe_3_ is *trans* to a Cl. The octahedral coordination is completed with the 2nd Cl being *trans* to a CO ligand. In both compounds, the ligands around Fe lie in slightly distorted octahedral coordination geometry. An overlay plot of the two molecules drawn by *Mercury* (Macrae *et al.*, 2008) shows the close similarity of the two molecules (Fig. 2).

There are weak intermolecular interactions of the type C–H···O which are observed between both the carbonyl O atoms of (I) and a methyl hydrogen atom of (II). The O of the solvent THF also has weak interactions with a methyl hydrogen atoms of (II) (Table 1).

### Experimental

FeCl_2_ (0.21 g, 1.62 *x* 10 ^-3^ mol) and PMe_3_ (0.40 ml, 3.86 *x* 10^-3^) were stirred in 20 ml of THF for 10 min, producing a clear gray solution of Cl_2_Fe(PMe_3_)_2_ (Harris *et al.*, 1978) in the presence of excess PMe_3_. Carbon monoxide was then bubbled through the solution until the color changed to an intense orange. The THF solvent was removed under vacuum and the resulting powder was extracted with pentane. After filtration through Celite, the pentane was removed under vacuum. The product was dissolved in a 1:2 mixture of THF and pentane and cooled to 243 K, causing orange crystals to form overnight.

### Refinement

H atoms bonded to the C atoms located on the mirror planes were located in a difference map and refined using a riding model. Other H atoms were calculated with idealized geometries with C–H = 0.98 and 0.99 Å for methyl and methylene type H-atoms, respectively, and refined using a riding model with *U*_iso_(H) = 1.2 (1.5 for methyl groups) times *U*_eq_(C). A molecule of THF was located in the asymmetric unit wherein C4 was disordered with partial occupancy factors 0.633 (9) and 0.367 (9).

### Crystal data

**Table:**

[FeCl_2_(C_3_H_9_P)_3_(CO)]·[FeCl_2_(C_3_H_9_P)_2_(CO)_2_]·2C_4_H_8_O	*D*_x_ = 1.397 Mg m^−^^3^
*M**_r_* = 862.10	Mo *K*α radiation, λ = 0.71073 Å
Orthorhombic, *P**n**m**a*	Cell parameters from 9975 reflections
*a* = 10.8391 (9) Å	θ = 2.6–27.4°
*b* = 16.9670 (12) Å	µ = 1.20 mm^−^^1^
*c* = 22.2871 (18) Å	*T* = 100 K
*V* = 4098.8 (6) Å^3^	Plate, light yellow
*Z* = 4	0.20 × 0.12 × 0.10 mm
*F*(000) = 1808	

### Data collection

**Table:**

Bruker APEXII CCD diffractometer	4904 independent reflections
Radiation source: fine-focus sealed tube	3939 reflections with *I* > 2σ(*I*)
graphite	*R*_int_ = 0.090
Detector resolution: 8.3333 pixels mm^-1^	θ_max_ = 27.6°, θ_min_ = 1.5°
φ and ω scans	*h* = −14→14
Absorption correction: multi-scan (*SADABS*; Bruker, 2008)	*k* = −21→22
*T*_min_ = 0.800, *T*_max_ = 0.895	*l* = −29→29
144514 measured reflections	

### Refinement

**Table:**

Refinement on *F*^2^	Primary atom site location: structure-invariant direct methods
Least-squares matrix: full	Secondary atom site location: difference Fourier map
*R*[*F*^2^ > 2σ(*F*^2^)] = 0.034	Hydrogen site location: inferred from neighbouring sites
*wR*(*F*^2^) = 0.081	H-atom parameters constrained
*S* = 1.06	*w* = 1/[σ^2^(*F*_o_^2^) + (0.0323*P*)^2^ + 5.017*P*] where *P* = (*F*_o_^2^ + 2*F*_c_^2^)/3
4904 reflections	(Δ/σ)_max_ = 0.002
218 parameters	Δρ_max_ = 0.68 e Å^−^^3^
1 restraint	Δρ_min_ = −0.70 e Å^−^^3^

### Special details

**Table:**

Geometry. All e.s.d.'s (except the e.s.d. in the dihedral angle between two l.s. planes) are estimated using the full covariance matrix. The cell e.s.d.'s are taken into account individually in the estimation of e.s.d.'s in distances, angles and torsion angles; correlations between e.s.d.'s in cell parameters are only used when they are defined by crystal symmetry. An approximate (isotropic) treatment of cell e.s.d.'s is used for estimating e.s.d.'s involving l.s. planes.
Refinement. Refinement of *F*^2^ against ALL reflections. The weighted *R*-factor *wR* and goodness of fit *S* are based on *F*^2^, conventional *R*-factors *R* are based on *F*, with *F* set to zero for negative *F*^2^. The threshold expression of *F*^2^ > σ(*F*^2^) is used only for calculating *R*-factors(gt) *etc*. and is not relevant to the choice of reflections for refinement. *R*-factors based on *F*^2^ are statistically about twice as large as those based on *F*, and *R*- factors based on ALL data will be even larger. *SHELX* restraints used:delu o2 c2

### Fractional atomic coordinates and isotropic or equivalent isotropic displacement parameters (Å^2^)

**Table:**

	*x*	*y*	*z*	*U*_iso_*/*U*_eq_	Occ. (<1)
Fe1	0.74441 (4)	0.2500	0.609986 (19)	0.01347 (10)	
Cl1	0.64447 (5)	0.14941 (3)	0.66198 (2)	0.02064 (12)	
P1	0.89145 (8)	0.2500	0.68214 (4)	0.01903 (18)	
P2	0.58528 (8)	0.2500	0.54398 (4)	0.01575 (17)	
O1	0.86416 (17)	0.37737 (10)	0.54685 (8)	0.0268 (4)	
C1	0.8188 (2)	0.32598 (13)	0.57122 (10)	0.0180 (5)	
C11	0.9949 (3)	0.33405 (17)	0.68026 (13)	0.0353 (7)	
H11A	0.9465	0.3828	0.6812	0.053*	
H11B	1.0441	0.3324	0.6434	0.053*	
H11C	1.0498	0.3323	0.7152	0.053*	
C12	0.8343 (4)	0.2500	0.75831 (15)	0.0277 (8)	
H12A	0.7861	0.2036	0.7650	0.042*	
H12B	0.9084	0.2500	0.7829	0.042*	
C21	0.5823 (2)	0.33398 (15)	0.49372 (12)	0.0258 (5)	
H21A	0.5116	0.3294	0.4665	0.039*	
H21B	0.6587	0.3354	0.4703	0.039*	
H21C	0.5749	0.3826	0.5172	0.039*	
C22	0.4336 (3)	0.2500	0.57645 (16)	0.0263 (8)	
H22A	0.3697	0.2500	0.5460	0.039*	
H22B	0.4224	0.2044	0.6013	0.039*	
Fe2	0.39182 (4)	0.7500	0.91114 (2)	0.01534 (11)	
Cl2	0.17671 (7)	0.7500	0.90064 (4)	0.02288 (17)	
Cl3	0.38648 (9)	0.7500	1.01832 (4)	0.0306 (2)	
P3	0.43591 (8)	0.7500	0.81372 (4)	0.01842 (18)	
P4	0.36847 (6)	0.61635 (3)	0.91539 (3)	0.01807 (13)	
O2	0.6473 (3)	0.7500	0.92770 (12)	0.0318 (6)	
C2	0.5567 (5)	0.7500	0.92171 (15)	0.0287 (9)	
C31	0.3099 (3)	0.7500	0.76165 (16)	0.0280 (8)	
H31A	0.3440	0.7500	0.7207	0.042*	
H31B	0.2601	0.7974	0.7654	0.042*	
C32	0.5302 (2)	0.66852 (14)	0.78642 (11)	0.0229 (5)	
H32A	0.4848	0.6189	0.7912	0.034*	
H32B	0.6071	0.6661	0.8095	0.034*	
H32C	0.5493	0.6767	0.7439	0.034*	
C41	0.2881 (3)	0.58171 (15)	0.98174 (11)	0.0291 (6)	
H41A	0.2086	0.6088	0.9851	0.044*	
H41B	0.3381	0.5929	1.0174	0.044*	
H41C	0.2743	0.5248	0.9786	0.044*	
C42	0.2761 (2)	0.56933 (14)	0.85754 (11)	0.0247 (5)	
H42A	0.2698	0.5128	0.8659	0.037*	
H42B	0.3152	0.5772	0.8184	0.037*	
H42C	0.1933	0.5926	0.8572	0.037*	
C43	0.5079 (2)	0.55672 (14)	0.91735 (11)	0.0240 (5)	
H43A	0.5573	0.5712	0.9525	0.036*	
H43B	0.5560	0.5659	0.8808	0.036*	
H43C	0.4855	0.5009	0.9198	0.036*	
C1S	0.7599 (3)	0.48598 (17)	0.79495 (13)	0.0420 (7)	
H1S1	0.7804	0.4575	0.7575	0.050*	
H1S2	0.6691	0.4908	0.7978	0.050*	
C2S	0.8096 (3)	0.44219 (17)	0.84835 (14)	0.0413 (7)	
H2S1	0.7417	0.4181	0.8717	0.050*	
H2S2	0.8672	0.4001	0.8355	0.050*	
C3S	0.8759 (3)	0.50294 (18)	0.88488 (13)	0.0400 (7)	
H3S1	0.8205	0.5280	0.9146	0.048*	0.633 (9)
H3S2	0.9481	0.4801	0.9059	0.048*	0.633 (9)
C4S	0.9139 (6)	0.5590 (4)	0.8379 (3)	0.0371 (13)	0.633 (9)
H4S1	0.9280	0.6118	0.8555	0.044*	0.633 (9)
H4S2	0.9912	0.5411	0.8186	0.044*	0.633 (9)
O1S	0.8150 (2)	0.56183 (12)	0.79471 (9)	0.0454 (6)	
H3S3	0.9638	0.4884	0.8897	0.048*	0.367 (9)
H3S4	0.8382	0.5075	0.9252	0.048*	0.367 (9)
C4S'	0.8641 (11)	0.5827 (7)	0.8499 (5)	0.0371 (13)	0.367 (9)
H4S3	0.8085	0.6195	0.8713	0.044*	0.367 (9)
H4S4	0.9457	0.6080	0.8450	0.044*	0.367 (9)

### Atomic displacement parameters (Å^2^)

**Table:**

	*U*^11^	*U*^22^	*U*^33^	*U*^12^	*U*^13^	*U*^23^
Fe1	0.0166 (2)	0.0124 (2)	0.0114 (2)	0.000	−0.00049 (17)	0.000
Cl1	0.0240 (3)	0.0195 (3)	0.0184 (3)	−0.0034 (2)	0.0011 (2)	0.0051 (2)
P1	0.0192 (4)	0.0221 (4)	0.0157 (4)	0.000	−0.0032 (3)	0.000
P2	0.0188 (4)	0.0154 (4)	0.0130 (4)	0.000	−0.0015 (3)	0.000
O1	0.0345 (10)	0.0197 (9)	0.0262 (9)	−0.0062 (7)	0.0047 (8)	0.0025 (7)
C1	0.0199 (11)	0.0182 (11)	0.0159 (11)	0.0019 (9)	−0.0024 (9)	−0.0033 (9)
C11	0.0331 (15)	0.0431 (16)	0.0295 (14)	−0.0166 (12)	−0.0103 (12)	0.0045 (12)
C12	0.032 (2)	0.036 (2)	0.0151 (17)	0.000	−0.0034 (15)	0.000
C21	0.0278 (13)	0.0243 (13)	0.0253 (13)	0.0000 (10)	−0.0057 (10)	0.0076 (10)
C22	0.0202 (17)	0.035 (2)	0.0233 (18)	0.000	−0.0009 (14)	0.000
Fe2	0.0170 (2)	0.0139 (2)	0.0151 (2)	0.000	0.00190 (18)	0.000
Cl2	0.0173 (4)	0.0231 (4)	0.0282 (4)	0.000	0.0019 (3)	0.000
Cl3	0.0299 (5)	0.0303 (5)	0.0314 (5)	0.000	0.0029 (4)	0.000
P3	0.0199 (4)	0.0185 (4)	0.0168 (4)	0.000	−0.0012 (3)	0.000
P4	0.0214 (3)	0.0147 (3)	0.0181 (3)	−0.0008 (2)	0.0026 (2)	0.0005 (2)
O2	0.0430 (17)	0.0244 (14)	0.0281 (15)	0.000	0.0089 (13)	0.000
C2	0.066 (3)	0.0088 (15)	0.0116 (16)	0.000	0.0014 (18)	0.000
C31	0.0295 (19)	0.0314 (19)	0.0231 (18)	0.000	−0.0049 (15)	0.000
C32	0.0262 (12)	0.0209 (12)	0.0216 (12)	0.0000 (9)	0.0044 (10)	−0.0014 (9)
C41	0.0387 (15)	0.0201 (12)	0.0284 (13)	−0.0003 (11)	0.0112 (12)	0.0053 (10)
C42	0.0257 (13)	0.0197 (12)	0.0287 (13)	−0.0063 (10)	−0.0027 (10)	−0.0017 (10)
C43	0.0267 (13)	0.0182 (12)	0.0272 (13)	0.0027 (10)	−0.0013 (10)	−0.0005 (10)
C1S	0.066 (2)	0.0282 (15)	0.0321 (15)	−0.0039 (14)	−0.0086 (15)	−0.0017 (12)
C2S	0.055 (2)	0.0303 (15)	0.0383 (17)	−0.0008 (14)	−0.0094 (15)	0.0058 (13)
C3S	0.0549 (19)	0.0383 (17)	0.0269 (14)	−0.0017 (14)	−0.0064 (14)	0.0052 (12)
C4S	0.040 (4)	0.042 (3)	0.029 (3)	−0.013 (2)	−0.008 (2)	0.005 (2)
O1S	0.0659 (15)	0.0324 (11)	0.0378 (12)	−0.0139 (10)	−0.0191 (11)	0.0110 (9)
C3S'	0.0549 (19)	0.0383 (17)	0.0269 (14)	−0.0017 (14)	−0.0064 (14)	0.0052 (12)
C4S'	0.040 (4)	0.042 (3)	0.029 (3)	−0.013 (2)	−0.008 (2)	0.005 (2)
O1S'	0.0659 (15)	0.0324 (11)	0.0378 (12)	−0.0139 (10)	−0.0191 (11)	0.0110 (9)

### Geometric parameters (Å, °)

**Table:**

Fe1—C1^i^	1.749 (2)	P4—C42	1.817 (2)
Fe1—C1	1.749 (2)	P4—C43	1.819 (2)
Fe1—P1	2.2641 (10)	O2—C2	0.991 (5)
Fe1—P2	2.2670 (9)	C31—H31A	0.9837
Fe1—Cl1^i^	2.3300 (6)	C31—H31B	0.9732
Fe1—Cl1	2.3300 (6)	C32—H32A	0.9800
P1—C12	1.807 (4)	C32—H32B	0.9800
P1—C11	1.814 (3)	C32—H32C	0.9800
P1—C11^i^	1.814 (3)	C41—H41A	0.9800
P2—C22	1.796 (4)	C41—H41B	0.9800
P2—C21	1.813 (2)	C41—H41C	0.9800
P2—C21^i^	1.813 (2)	C42—H42A	0.9800
O1—C1	1.139 (3)	C42—H42B	0.9800
C11—H11A	0.9800	C42—H42C	0.9800
C11—H11B	0.9800	C43—H43A	0.9800
C11—H11C	0.9800	C43—H43B	0.9800
C12—H12A	0.9565	C43—H43C	0.9800
C12—H12B	0.9731	C1S—O1S	1.419 (3)
C21—H21A	0.9800	C1S—C2S	1.503 (4)
C21—H21B	0.9800	C1S—H1S1	0.9899
C21—H21C	0.9800	C1S—H1S2	0.9896
C22—H22A	0.9693	C2S—C3S	1.497 (4)
C22—H22B	0.9597	C2S—H2S1	0.9895
Fe2—C2	1.803 (5)	C2S—H2S2	0.9908
Fe2—P3	2.2232 (10)	C3S—C4S	1.472 (6)
Fe2—P4^ii^	2.2837 (6)	C3S—H3S1	0.9900
Fe2—P4	2.2838 (6)	C3S—H3S2	0.9900
Fe2—Cl2	2.3433 (9)	C4S—O1S	1.442 (6)
Fe2—Cl3	2.3895 (11)	C4S—H4S1	0.9900
P3—C31	1.792 (4)	C4S—H4S2	0.9900
P3—C32	1.823 (2)	C4S'—H4S3	0.9900
P3—C32^ii^	1.823 (2)	C4S'—H4S4	0.9900
P4—C41	1.814 (2)		
			
C1^i^—Fe1—C1	94.97 (15)	C31—P3—Fe2	117.95 (13)
C1^i^—Fe1—P1	91.50 (8)	C32—P3—Fe2	116.50 (8)
C1—Fe1—P1	91.50 (8)	C32^ii^—P3—Fe2	116.50 (8)
C1^i^—Fe1—P2	91.74 (8)	C41—P4—C42	99.88 (13)
C1—Fe1—P2	91.74 (8)	C41—P4—C43	101.48 (12)
P1—Fe1—P2	175.20 (4)	C42—P4—C43	103.34 (12)
C1^i^—Fe1—Cl1^i^	179.61 (8)	C41—P4—Fe2	114.12 (9)
C1—Fe1—Cl1^i^	85.42 (7)	C42—P4—Fe2	117.88 (8)
P1—Fe1—Cl1^i^	88.51 (2)	C43—P4—Fe2	117.46 (8)
P2—Fe1—Cl1^i^	88.22 (2)	O2—C2—Fe2	179.8 (4)
C1^i^—Fe1—Cl1	85.42 (7)	P3—C31—H31A	108.3
C1—Fe1—Cl1	179.61 (8)	P3—C31—H31B	111.6
P1—Fe1—Cl1	88.51 (2)	H31A—C31—H31B	106.7
P2—Fe1—Cl1	88.22 (2)	P3—C32—H32A	109.5
Cl1^i^—Fe1—Cl1	94.19 (3)	P3—C32—H32B	109.5
C12—P1—C11	103.51 (12)	H32A—C32—H32B	109.5
C12—P1—C11^i^	103.51 (12)	P3—C32—H32C	109.5
C11—P1—C11^i^	103.6 (2)	H32A—C32—H32C	109.5
C12—P1—Fe1	115.20 (13)	H32B—C32—H32C	109.5
C11—P1—Fe1	114.74 (9)	P4—C41—H41A	109.5
C11^i^—P1—Fe1	114.74 (9)	P4—C41—H41B	109.5
C22—P2—C21	103.45 (11)	H41A—C41—H41B	109.5
C22—P2—C21^i^	103.45 (11)	P4—C41—H41C	109.5
C21—P2—C21^i^	103.62 (18)	H41A—C41—H41C	109.5
C22—P2—Fe1	115.78 (12)	H41B—C41—H41C	109.5
C21—P2—Fe1	114.50 (9)	P4—C42—H42A	109.5
C21^i^—P2—Fe1	114.50 (9)	P4—C42—H42B	109.5
O1—C1—Fe1	177.4 (2)	H42A—C42—H42B	109.5
P1—C11—H11A	109.5	P4—C42—H42C	109.5
P1—C11—H11B	109.5	H42A—C42—H42C	109.5
H11A—C11—H11B	109.5	H42B—C42—H42C	109.5
P1—C11—H11C	109.5	P4—C43—H43A	109.5
H11A—C11—H11C	109.5	P4—C43—H43B	109.5
H11B—C11—H11C	109.5	H43A—C43—H43B	109.5
P1—C12—H12A	109.5	P4—C43—H43C	109.5
P1—C12—H12B	104.3	H43A—C43—H43C	109.5
H12A—C12—H12B	111.3	H43B—C43—H43C	109.5
P2—C21—H21A	109.5	O1S—C1S—C2S	107.5 (2)
P2—C21—H21B	109.5	O1S—C1S—H1S1	110.2
H21A—C21—H21B	109.5	C2S—C1S—H1S1	110.2
P2—C21—H21C	109.5	O1S—C1S—H1S2	110.1
H21A—C21—H21C	109.5	C2S—C1S—H1S2	110.3
H21B—C21—H21C	109.5	H1S1—C1S—H1S2	108.5
P2—C22—H22A	111.9	C3S—C2S—C1S	105.2 (2)
P2—C22—H22B	110.4	C3S—C2S—H2S1	110.8
H22A—C22—H22B	108.2	C1S—C2S—H2S1	110.7
C2—Fe2—P3	85.09 (11)	C3S—C2S—H2S2	110.5
C2—Fe2—P4^ii^	96.00 (2)	C1S—C2S—H2S2	110.7
P3—Fe2—P4^ii^	93.689 (19)	H2S1—C2S—H2S2	108.9
C2—Fe2—P4	96.00 (2)	C4S—C3S—C2S	101.1 (3)
P3—Fe2—P4	93.687 (19)	C4S—C3S—H3S1	111.6
P4^ii^—Fe2—P4	166.40 (4)	C2S—C3S—H3S1	111.6
C2—Fe2—Cl2	178.23 (11)	C4S—C3S—H3S2	111.6
P3—Fe2—Cl2	96.68 (4)	C2S—C3S—H3S2	111.6
P4^ii^—Fe2—Cl2	83.905 (19)	H3S1—C3S—H3S2	109.4
P4—Fe2—Cl2	83.906 (19)	O1S—C4S—C3S	106.8 (4)
C2—Fe2—Cl3	83.88 (11)	O1S—C4S—H4S1	110.4
P3—Fe2—Cl3	168.98 (4)	C3S—C4S—H4S1	110.4
P4^ii^—Fe2—Cl3	87.469 (19)	O1S—C4S—H4S2	110.4
P4—Fe2—Cl3	87.471 (19)	C3S—C4S—H4S2	110.4
Cl2—Fe2—Cl3	94.34 (3)	H4S1—C4S—H4S2	108.6
C31—P3—C32	102.17 (11)	C1S—O1S—C4S	106.3 (3)
C31—P3—C32^ii^	102.17 (11)	H4S3—C4S'—H4S4	108.9
C32—P3—C32^ii^	98.60 (16)		
			
C1^i^—Fe1—P1—C12	−132.49 (7)	P4^ii^—Fe2—P3—C32	153.62 (10)
C1—Fe1—P1—C12	132.49 (7)	P4—Fe2—P3—C32	−37.81 (10)
Cl1^i^—Fe1—P1—C12	47.118 (16)	Cl2—Fe2—P3—C32	−122.09 (9)
Cl1—Fe1—P1—C12	−47.117 (16)	Cl3—Fe2—P3—C32	57.91 (9)
C1^i^—Fe1—P1—C11	107.43 (14)	C2—Fe2—P3—C32^ii^	−57.91 (9)
C1—Fe1—P1—C11	12.42 (14)	P4^ii^—Fe2—P3—C32^ii^	37.81 (10)
Cl1^i^—Fe1—P1—C11	−72.95 (12)	P4—Fe2—P3—C32^ii^	−153.62 (9)
Cl1—Fe1—P1—C11	−167.19 (12)	Cl2—Fe2—P3—C32^ii^	122.09 (9)
C1^i^—Fe1—P1—C11^i^	−12.42 (14)	Cl3—Fe2—P3—C32^ii^	−57.91 (9)
C1—Fe1—P1—C11^i^	−107.43 (14)	C2—Fe2—P4—C41	111.10 (15)
Cl1^i^—Fe1—P1—C11^i^	167.19 (12)	P3—Fe2—P4—C41	−163.46 (11)
Cl1—Fe1—P1—C11^i^	72.96 (12)	P4^ii^—Fe2—P4—C41	−40.7 (2)
C1^i^—Fe1—P2—C22	132.49 (7)	Cl2—Fe2—P4—C41	−67.12 (11)
C1—Fe1—P2—C22	−132.49 (7)	Cl3—Fe2—P4—C41	27.52 (11)
Cl1^i^—Fe1—P2—C22	−47.128 (16)	C2—Fe2—P4—C42	−132.18 (15)
Cl1—Fe1—P2—C22	47.126 (16)	P3—Fe2—P4—C42	−46.74 (10)
C1^i^—Fe1—P2—C21	−107.25 (12)	P4^ii^—Fe2—P4—C42	76.00 (19)
C1—Fe1—P2—C21	−12.23 (12)	Cl2—Fe2—P4—C42	49.60 (10)
Cl1^i^—Fe1—P2—C21	73.13 (10)	Cl3—Fe2—P4—C42	144.24 (10)
Cl1—Fe1—P2—C21	167.38 (10)	C2—Fe2—P4—C43	−7.49 (15)
C1^i^—Fe1—P2—C21^i^	12.23 (12)	P3—Fe2—P4—C43	77.96 (10)
C1—Fe1—P2—C21^i^	107.25 (12)	P4^ii^—Fe2—P4—C43	−159.30 (17)
Cl1^i^—Fe1—P2—C21^i^	−167.39 (10)	Cl2—Fe2—P4—C43	174.30 (10)
Cl1—Fe1—P2—C21^i^	−73.14 (10)	Cl3—Fe2—P4—C43	−91.06 (10)
C2—Fe2—P3—C31	180.0	O1S—C1S—C2S—C3S	11.0 (4)
P4^ii^—Fe2—P3—C31	−84.29 (2)	C1S—C2S—C3S—C4S	−28.5 (4)
P4—Fe2—P3—C31	84.29 (2)	C2S—C3S—C4S—O1S	36.8 (5)
Cl2—Fe2—P3—C31	0.0	C2S—C1S—O1S—C4S	11.8 (4)
Cl3—Fe2—P3—C31	180.000 (1)	C3S—C4S—O1S—C1S	−31.1 (6)
C2—Fe2—P3—C32	57.91 (9)		

Symmetry codes: (i) *x*, −*y*+1/2, *z*; (ii) *x*, −*y*+3/2, *z*.

### Hydrogen-bond geometry (Å, °)

**Table:**

*D*—H···*A*	*D*—H	H···*A*	*D*···*A*	*D*—H···*A*
C42—H42B···O1S^iii^	0.98	2.53	3.422 (3)	151
C43—H43C···O1^iii^	0.98	2.58	3.510 (3)	158
C43—H43A···O1^iv^	0.98	2.43	3.392 (3)	167

Symmetry codes: (iii) *x*−1/2, *y*, −*z*+3/2; (iv) −*x*+3/2, −*y*+1, *z*+1/2.
